# Facilitators to and experience of psychological resilience during disease response among people with diabetes: a mixed-methods study using resilience framework

**DOI:** 10.3389/fpsyg.2026.1796873

**Published:** 2026-06-23

**Authors:** Hantian Cheng, Tianhong Wen, Yang Lei, Zheng Lin, Qingyu Wang, Jiali Chen, Yanzhe Wang, Xiaorui Tang

**Affiliations:** School of Nursing, Nanjing Medical University, Nanjing, China

**Keywords:** diabetes mellitus, psychological resilience, facilitators and experience, mixed-methods study, descriptive phenomenological research

## Abstract

**Background:**

In people with diabetes, strong psychological resilience can help them effectively adapt to the lifestyle changes and emotional fluctuations caused by the disease. Integrating quantitative research (identifying key factors and their statistical associations) and qualitative research (exploring in-depth individual experiences) can provide a more comprehensive understanding of resilience facilitators, which has important implications for integrating resilience interventions into future psychological care practices.

**Objective:**

Informed by the resilience framework, this study elucidates the factors contributing to the development of a positive level of psychological resilience and examines how/why people responded to diabetes.

**Design:**

Convergent mixed-methods study with a phenomenological qualitative component.

**Methods:**

This mixed-methods study combined a cross-sectional survey (*N* = 290) with in-depth interviews (*N* = 15) in two hospitals in China (Sep–Nov 2023). Quantitatively, standardized questionnaires assessed psychological resilience in adults (≥18y) with physician-diagnosed diabetes. Qualitatively, phenomenological interviews explored post-diagnosis psychological experiences and resilience facilitators guided by Kumpfer's resilience framework. Regression and thematic analyses were applied, with joint displays for integration.

**Results:**

A total of 290 people with diabetes completed the survey questionnaires, and 15 completed an interview. From the environmental context, social support (β = 0.34, *p* < 0.001) and social resources accessibility enhanced resilience. Regarding internal resiliency factors, self-efficacy (β = 0.99, *p* < 0.001) and positive mindset emerged as strong positive correlates, while depression (β = –29.31, *p* < 0.001) constituted a significant barrier. Notably, qualitative research revealed a novel dimension of “Altruism”—many patients cited responsibility for their children's health as a driving force. Additionally, Qualitative data added “diabetes learning journey” and “physiological reserve” as internal resilience resources. The most compelling findings emerged from Person-environmental transactional process: quantitative data showed avoidance coping to be ineffective (*p* = 0.81). Acceptance coping showed paradox: harmful quantitatively (β = –0.98) but helpful qualitatively. This discrepancy likely stems from the fundamental difference between “adaptive acceptance” vs. “passive surrender.” Both cognitive reappraisal and the power of role models promoted resilience. The most crucial integrative finding reveals a bidirectional reinforcement between psychological resilience and self-efficacy, challenging traditional unidirectional models.

**Conclusions:**

Optimizing resilience facilitators while addressing barriers is likely to be associated with higher psychological resilience in diabetes patients. Key findings demonstrate the importance of expanding social support networks and promoting resources access, empowering patients through positive mindset building and cognitive reappraisal techniques (e.g., humor coping, benefit-finding), and implementing motivational strategies anchored in altruism (e.g., family health goals). The study particularly underscores the need to differentiate adaptive acceptance from passive surrender in coping processes. In clinical and psychological practice, dynamic monitoring tools should be integrated to quantify the dual effects of acceptance coping and track the bidirectional reinforcement cycle between resilience and self-efficacy. Systematically incorporating these strategies into chronic disease health education and psychological interventions may be beneficial for both mental health and disease management.

## 1 Introduction

Diabetes is one of the most common chronic non-communicable diseases, which is a major global cause of morbidity and mortality (Ong et al., 2023). The International Diabetes Federation estimated that in 2024, there were 5.89 million people worldwide living with diabetes, leading to a global health expenditure of 1,000 billion dollars and by 2050, the number of affected individuals would reach 8.53 million (Genitsaridi et al., 2026).

Diabetes has a long course and is prone to multiple systemic damages, such as retinopathy, chronic kidney disease, cardiovascular diseases, and amputation, which severely affect the quality of life (Park et al., 2022; Bohler et al., 2024; Avogaro and Fadini, 2019). The patients need lifelong self-management, which is a demanding and challenging task (Perrin et al., 2017) to bring a significant impact on patients' mental wellbeing. People are prone to experiencing various negative emotions when coping with diabetes, such as depression, anxiety, and chronic stress from the demands of rigorous self-management (Perrin et al., 2017; Chew et al., 2017; Hessler et al., 2017). All these issues might present barriers to the patients' adherence to diet, exercise, medication prescriptions, thereby negatively affecting the overall treatment outcomes (Sachar et al., 2020; Hoogendoorn et al., 2024). An extensive survey on the psychological wellbeing and health among people with diabetes, titled “Too often missing,” revealed that 70% of people with diabetes felt overwhelmed by their condition, with three-quarters of them stating that their self-management was affected by emotional struggles (UK D, 2019).

However, despite significant challenges, some resilient people with diabetes are able to adhere to recommended diabetes care behaviors, achieve blood glucose targets, and reduce distress (Mei et al., 2023). This prompts researchers to consider the psychological impact of diabetes on patients from a positive and active perspective. Psychologists define psychological resilience as the process of positive adaption in the face of adversity, trauma, tragedy, threat, or other threatening context, which refers to individual's capacity to bounce back from adversity and involves profound personal growth (Anderson and Priebe, 2021; Dai et al., 2024).

The resilience was positively associated with adherence to diabetes self-management behaviors among African-American males residing in diabetes hotspots (Jia et al., 2022). Furthermore, psychological resilience can predict future HbA1c and buffer against worsening HbA1c and self-management when levels of distress increase in people with diabetes (Pesantes et al., 2015; Yi-Frazier et al., 2024). Additionally, promoting psychological resilience was an effective way to alleviate stress and improve self-efficacy among adolescent diabetes patients (Wu et al., 2023). It is evident that enhanced levels of psychological resilience are of significant importance in mitigating the progression of the disease.

Similar to building a muscle, an individual's psychological resilience is malleable (Linz et al., 2020; Kunzler et al., 2020). The mechanism of promoting interventions for psychological resilience mainly involves improving facilitators of people with diabetes (Joyce et al., 2018). To date, several factors promoting resilience have already been explored. In a multicenter study on diabetes, increased levels of physical activity were found to promote greater psychological resilience in patients with type 1 diabetes (Lukács et al., 2018). Qualitative interviews with 62 emerging adults diabetes were conducted and three protective factors were identified: interpersonal strategies, positive cognition, and coping with challenges (Skedgell et al., 2021). A study targeting adolescents with diabetes showed that engagement coping strategies was associated with higher psychological resilience (Straton et al., 2024). Although type 1 and type 2 diabetes differ in etiology, both entail lifelong self-management stress and fear of complications (Wang et al., 2023; Yi-Frazier et al., 2024). Therefore, resilience-building strategies are equally essential for all types.

Relevant studies have attempted to identify facilitators of psychological resilience using a single research design, but there remains a gap in the effectiveness among people with diabetes. No consensus or definitive guidance exists on which resilience aspects require action (Pesantes et al., 2015). When researchers qualitatively study the psychological resilience of a few people with diabetes, they lost the ability to generalize the results to many (Kusnanto et al., 2020). Also, that previous study sampled a narrow age group (emerging adults) and may not be generalizable to the broader adult diabetes population (Skedgell et al., 2021). When conducting quantitative research on many people with diabetes, the understanding of any individual may be reduced (Rivera-Picón et al., 2023). Previous quantitative research has primarily focused on measuring coping strategies and external resources, with less exploration of individual factors. Mixed research is the type of study in which researchers combine elements of qualitative and quantitative research methods with the aim of achieving breadth and depth of understanding and corroboration (Guetterman et al., 2015). It provides a way to leverage strengths to offset the weaknesses of quantitative and qualitative studies and can offer deep insights of facilitators of psychological resilience beyond the sum of single research method (Palinkas et al., 2019).

Therefore, to address the current literature gaps, this mixed-methods study, based on Kumpfer's resilience framework and using a convergent design to determine the factors contributing to the resilience in people with diabetes. Kumpfer's resilience framework is based on an interactive social-ecological model and an individual-process-context model guided by a systems perspective, which has been widely applied in the research field of psychological resilience (Guo et al., 2022; Luo et al., 2019a). Kumpfer pointed out that facilitators and protective processes occurred at the individual, person-environment transactional (the ongoing interaction between person and environment), and environmental levels, demonstrating strong integrative nature (Yu et al., 2024).

## 2 Aim of the study

Specifically, this article aims to address the following questions: (a) What is the experience of psychological resilience processes in people with diabetes when managing their condition? (b) Based on individual, person-environment transactional, and environmental levels, what are the facilitators influencing psychological resilience?

## 3 Materials and methods

### 3.1 Design

**Figure 1:**
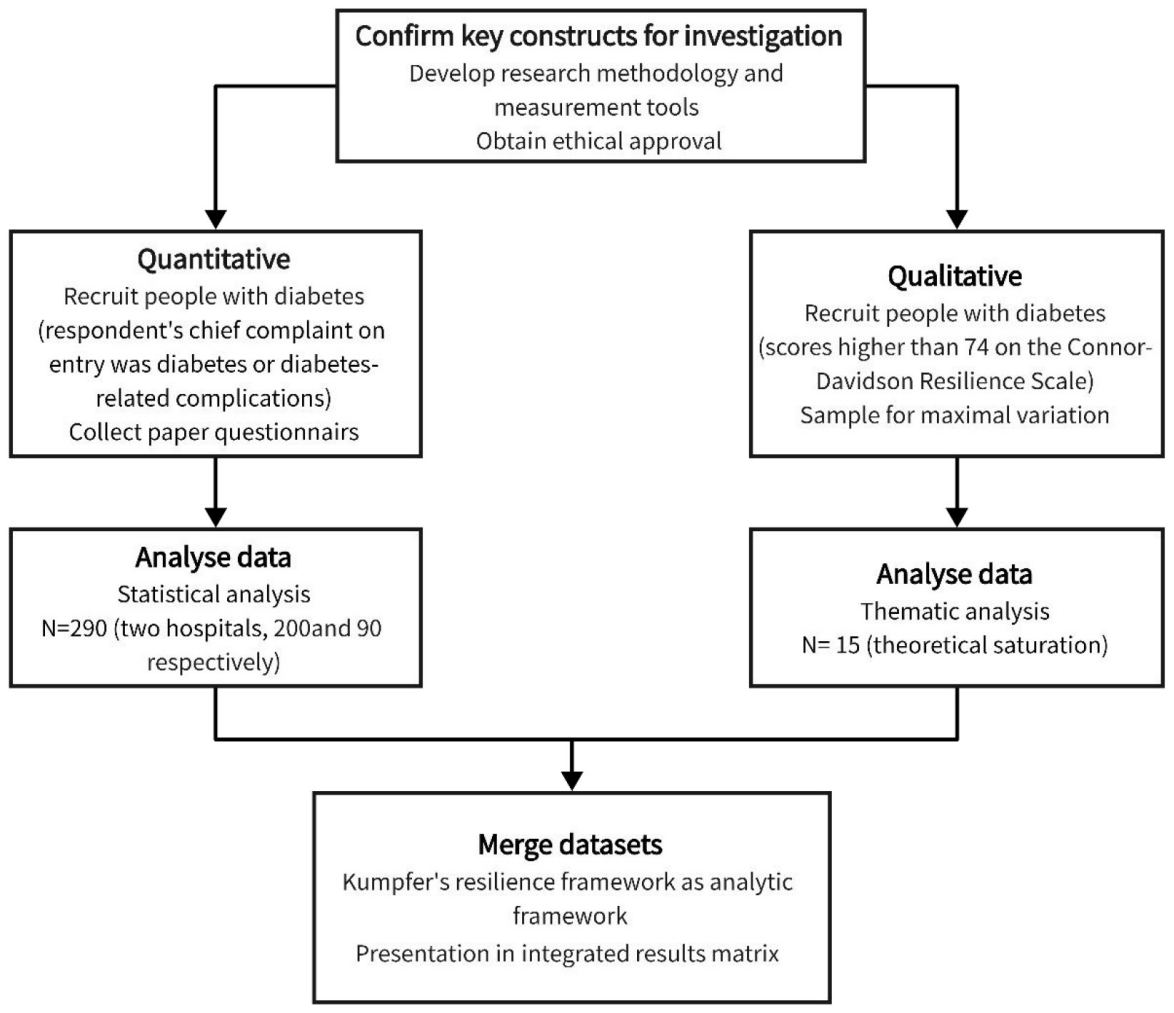
The convergent design of mixed-methods research.

A mixed-methods study, with a convergent design, was employed to synthesize complementary quantitative and qualitative findings. The quantitative study took the form of a cross-sectional survey, while the qualitative study was an in-depth interview. This design assigned equal priority and weight to quantitative and qualitative research, conducting both types of studies simultaneously (Östlund et al., 2011). In the integration phase, this study utilizes tables to present the combined results of both quantitative and qualitative research (Moeller et al., 2016). The convergent design is illustrated by Figure 1.

The quantitative study was a cross-sectional survey, using standardized questionnaires to explore the current status of psychological resilience among people with diabetes, as well as the factors contributing to the resilience, based on the resilience framework created by Kumpfer (Yu et al., 2024). In the quantitative study, the first set of the independent variables were sociodemographic variables and diabetes related characteristics. In the second set of independent variables, according to the resilience framework, depression, self-efficacy, social support, family care, medical coping strategies, and overall wellbeing were included, whereas the dependent variable was psychological resilience.

In the qualitative study, a descriptive phenomenological approach was used. This study employed in-depth interviews to elicit information about the real psychological experiences of diabetes patients after diagnosis, further understand how/why people responded to diabetes and elucidate the factors contributing to the development of a positive level of psychological resilience based on the resilience framework (Yu et al., 2024). It was carried out at the same time as the scales were collected.

### 3.2 Participants

This study employed a mixed-methods design. The quantitative and qualitative phases shared the same target population: people with diabetes admitted to the endocrine wards of two affiliated hospitals of Nanjing Medical University. All potential participants were first required to meet the quantitative inclusion criteria: (1) participants were aged 18 years or over, and (2) were diagnosed as type 1 diabetes or type 2 diabetes (confirmed by an endocrinology specialist and in accordance with international guidelines), (3) the willing respondents were also required to possess adequate Chinese language proficiency to complete written questionnaires and participate in interviews, (4) it was of utmost importance that the respondent's chief complaint on entry was diabetes or diabetes-related complications to ensure that diabetes was a major source of psychological coping for the participants (Diabetes or its complications constituted ≥70% of the primary health issues recorded in medical records). In order to achieve these objectives, the review of participants' case files by the nurse responsible for the participant's illness on each ward had assisted in screening the inclusion criteria. Interested participants were offered written informed consent and patients with diabetes having no interested in participating were excluded from the study.

On the basis of this quantitative sample, purposive sampling was used for the qualitative component, with participants selected based on scores higher than 74 on the Connor-Davidson Resilience Scale. A cut-off of >74 was chosen because it represents the 75th percentile in our sample. The maximum variation sampling strategy was employed to ensure diversity in terms of age, gender, level of education, and diabetes-related characteristics, to avoid potential biases resulting from the sample being limited to a specific population. The resilience score served as a crucial selection criterion to enable a better understanding of the factors promoting psychological resilience through the interviews. A total of 15 individual in-depth interviews were conducted; sample size was determined by data saturation, defined as no new themes emerging in three consecutive interviews.

### 3.3 Instruments

The quantitative study using standardized case report form collected data on the socio-demographic information and diabetic profile. In accordance with Kumpfer's resilience framework, the study employed the following scales to measure corresponding constructs: The Social Support Rating Scale (SSRS) and Family APGAR Index assessed environmental context; the Self-Rating Depression Scale (SDS), General Self-Efficacy Scale (GSES) and the General Wellbeing questionnaire measured internal resiliency factors; while the Medical Coping Modes Questionnaire (MCMQ) evaluated person-environment transactional process.

The Connor-Davidson Resilience Scale (CD-RISC) was used to measure psychological resilience in respondents, developed by Connor and Davidson in 2003 (Connor and Davidson, 2003). High scores were indicative of high levels of psychological resilience in the respondents. A 0.915 reliability coefficient was obtained in the current study for the CD-RISC.

The SDS authored by (Zung 1965) was used to measure the severity of depressive symptoms. Twenty items were on the scale, with a total score of 80 and high scores were translated as high levels of depression and vice versa. Depression Severity Index = Cumulative score/80. The scale has good reliability and validity, with a reliability coefficient of 0.793 in this study.

The GSES was developed by (Schwarzer and Jerusalem 2010) to assess an individual's overall confidence when facing challenges in different environments or encountering novel situations. It contained 10 items and a high level of self-efficiency was interpreted by a high score from respondents and vice versa. A 0.862 reliability coefficient was obtained in this study.

The Family APGAR Index, revised by (Zhang 2005), assesses satisfaction with family functioning in participants. High scores implied great functioning of the family and vice versa. The current study found a reliability coefficient of 0.804 for the APGAR.

The MCMQ authored by Fefeil Herman in 1991 (Feifel et al., 1987) is used to assess the patient's coping strategies toward the illness and consists of 20 terms containing three categories of coping styles: confrontation (8 terms), avoidance (7 terms) and surrender (5 terms). The reliability coefficient of the medical coping style questionnaire in this study was 0.498.

The SSRS developed by (Xiao 1994), was used to evaluate the objective support received, emotional support experienced, and utilization of support by participants. A reliability coefficient of 0.776 was obtained for the SSRS in the current study.

The General Wellbeing questionnaire was developed by the National Center for Health Statistics in the United States (Pouwer et al., 2000). A high score on this questionnaire meant that respondents had a high level of wellbeing. A 0.803 reliability coefficient was obtained in the current study.

In the qualitative study, a standardized interview guide was used (Table 1), developed from research group discussion and four domains of Kumpfer's resilience framework—environmental resources, internal resilience factors, person–environment transactional processes and resilience process—to ensure deep integration with the quantitative phase (Yu et al., 2024). This guide consisted of 10 open-ended questions relating how people feel about diabetes, the decisions they make when dealing with the troubles and frustrations caused by diabetes and the factors influencing those decisions. The focus of interviews was understanding facilitators of psychological resilience in patients' adaption to diabetes.

**Table 1:** Interview script.

Questions
1. How did you feel after learning about your illness? What do you think? Both physically and psychologically?
2. How have you dealt with these problems? Can you describe what efforts you have made or any ideas you have?
3. Have there been any changes in your psychological state throughout the progression of your illness?
4. What was your initial reaction when you were first diagnosed, and how has your psychological state changed since then? What adjustments have you made? How long did each stage last?
5. What elements in your living environment did you think have supported you in coping with the challenges of diabetes
6. What traits or abilities did you think helped you cope with the challenges of diabetes?
7. Could you share which coping strategies were particularly helpful for you in dealing with the challenges of diabetes?
8. What positive role did you think the strength or resilience you showed in facing diabetes played?
9. Have you experienced any changes in your psychological state during the course of your illness?
10. What do you think were the most important factors that helped you to recover during your illness?

### 3.4 Procedure

Participants were recruited by the first author over 2 months from endocrine inpatient wards in two affiliated hospitals of Nanjing Medical university in East China region (The First Affiliated Hospital of Nanjing Medical University and Xuanwu Hospital). On the basis of being informed, the willing participants need to sign a written informed consent form. Before the formal collection of questionnaires, a pre-test was conducted on 10 voluntary respondents with different socio-demographic backgrounds. These respondents were randomly selected from two hospitals, and all 10 participants were diagnosed with diabetes. They reported that these instrument were user-friendly. The formal data collection took place from September to November in 2023 via purposive sampling from endocrine wards, with no overlap from the pre-test. *A-priori* power analysis for multiple linear regression with G^*^Power 3.1 indicated a required sample size, leading to the enrolment of 290 people with diabetes (200 and 90, respectively). Four researchers conducted surveys using paper questionnaires and collected them immediately after completion, but they did not participate in the qualitative research. Quantitative questionnaires were completed anonymously and no personal identifiers were recorded.

For the qualitative phase, semi-structured personal interviews were conducted by each participant individually in a hospital lounge to ensure a relatively quiet environment. These interviews were carried out in the local language (Chinese) and audio-recorded. After the completion of the interviews, the recordings were translated into English, transcribed verbatim and cross-checked for quality and consistency by a second bilingual translator. Four members of the research team, who are trained in qualitative interviewing, were responsible for collecting and analyzing qualitative data, but they were not involved in the collection of experimental data and had no prior relationship with participants. The transcripts were read and re-read to identify likely and recurring themes. Audio files were encrypted, stored on a password-protected device accessible only to the research team, and destroyed after transcription.

### 3.5 Data analysis

The IBM SPSS statistics (v.26.0) was used for quantitative data analyses and *p* < 0.05 was considered statistically significant. For continuous variables, descriptive analysis was presented as mean ± standard deviation (SD), while for categorical variables, it was presented as frequency (percentages).

Multivariate linear regression (MLR) was used to estimate B [standard error (SE)] for the association of the independent variables with psychological resilience. Three models were generated for the analysis: Model 1 took socio-demographic variables and diabetes related characteristics as independent variables without adjusting for any variables; Model 2, based on Kumpfer's resilience framework, included family Apgar index, general self-efficacy, depression severity index, social support, surrender coping, confrontation coping and avoidance coping as independent variables, without adjusting for any variables. Model 3 adjusted for gender, age, level of education, region of residence hobbies, hobbies, family monthly income per capita, number of combined diseases or complications, and duration of diabetes based on Model 2. This study calculated the variance inflation factors (VIF) of all variables in the regression model to assess multicollinearity. A standard of VIF > 10 was used to indicate collinearity.

This study employed a combination of the resilience framework and deductive approaches to conduct thematic analysis of the qualitative data. All transcripts were independently reviewed and coded by four researchers to identify key emerging concepts. Colaizzi seven-step phenomenological method was used to guide the analysis of the data (Colaizzi, 1978). (i) Four researchers sufficiently familiarized themselves with the collected data through repeated and careful reading; (ii) Statements related to the true psychological experience of people with diabetes after diagnosis and the facilitators of psychological resilience were identified and extracted; (iii) The first author explicated the potential implications of these statements in relation to psychological resilience. To reduce bias, the research team conducted a bracketing interview to discuss the potential influence of preconceptions, biases, and past experiences that may influence the analysis process. These “brackets” were treated as data that were recorded and revisited throughout the analysis process (Powers and Knapp, 2006); (iv) After making adjustments based on the results of the bracketing interview, the researchers brought together the coded ideas, and once a consensus was reached among the entire research team, meaningful common concepts were developed into theme prototypes; (v) The researchers integrated the theme prototypes into the participants' detailed typical statements; (vi) The basic structure of the themes was created by comparing similar themes and descriptions, and then adjusting to include only the core and universal elements of psychological experience and the facilitators of psychological resilience; (vii) In the final step, the researcher returned the theme structure to the study participants, seeking validation of the results through a review of real experiences. A total of nine participants provided feedback indicating that their experiences were accurately captured. Nvivo 15.0 software was used for data storage, retrieval and coding.

### 3.6 Integration

Integration of quantitative data and qualitative data occurred in four steps (Creswell and Poth, 2018). Firstly, transforming the results of one of the databases into the other type of data was implemented, where factors that significantly affect psychological resilience from the quantitative phase were transformed into qualitative themes. Subsequently, the resilience framework domains of environmental characteristics, individual internal factors, and individual-environmental interaction process were used as an analytic framework to synthesize and interpret quantitative and qualitative findings. Finally, through the discussion, findings from each phase were integrated to gather a holistic understanding of factors for promoting the psychological resilience. This study integrated the holistic results into a narrative discussion is to specify how the qualitative quotes either confirm, disconfirm, or complement the quantitative results (Creswell and Creswell, 2018). “Convergence” occurs when narratives match the statistical trend; “divergence” when they highlight a variable the survey deems non-significant or opposite; “extension” when they introduce unmeasured themes that broaden the model. Joint displays are recommended for mixed-methods integration because they allow systematic comparison of quantitative and qualitative results, revealing patterns of convergence, divergence, and expansion that would otherwise remain implicit (Guetterman et al., 2015).

### 3.7 Ethical considerations

This study was approved by the Ethics Committee of Nanjing Medical University (Approval No. 2023-553). Informed assent and consent were secured from all participants involved in the study.

## 4 Results

### 4.1 Participant characteristics

290 participants were recruited (Table 2). The mean age was 53.98 ± 13.75 years. Most participants were male (57.9 %) with an average of 1.49 ± 1.30 co-morbidities or complications. A total of 60.7% of the participants suffered from diabetes for more than 3 years.

**Table 2:** Baseline characteristics of the participants according to psychological resilience status in the quantitative research.

Variable	Total	Psychological resilience score, Mean (SD)	*P*
Socio-demographic variables			
Gender, *n* (%)			0.020^*^
Male	168 (57.9)	65.37 (15.02)	
Female	122 (42.1)	61.48 (15.11)	
Age, year, Mean (SD)	53.98 (13.75)		0.444
Level of education, *n* (%)			< 0.001^*^
Primary school or less	56 (19.3)	54.59 (15.35)	
Junior high school	64 (22.1)	63.63 (14.79)	
Senior high school or secondary technical school	80 (27.6)	65.53 (14.31)	
Junior college	51 (17.6)	67.80 (12.88)	
Undergraduate and above	39 (13.4)	68.03 (15.12)	
Region of residence, *n* (%)			0.001^*^
Rural	99 (34.1)	59.77 (15.68)	
Urban	191 (65.9)	65.79 (14.49)	
Hobbies, *n* (%)			0.001^*^
Few	52 (17.9)	57.52 (14.80)	
A few	154 (53.1)	63.51 (14.81)	
Moderate	46 (15.9)	66.13 (15.60)	
Most	38 (13.1)	70.21 (13.58)	
Family monthly income per capita (RMB), *n* (%)			< 0.001^*^
< 500	31 (10.7)	54.06 (17.36)	
(500–1,000)	42 (14.5)	59.60 (15.62)	
(1,001–2,000)	41 (14.1)	62.82 (14.87)	
(2,001–3,000)	72 (24.8)	67.12 (12.51)	
>3,000	104 (35.9)	67.58 (13.83)	
Diabetes related characteristics			
Number of co-morbidities or complications, *n* (%)			0.003^*^
None	88 (30.3)	68.13 (15.53)	
1	71 (24.5)	59.03 (14.82)	
2	55 (19.0)	63.82 (14.54)	
3	54 (18.6)	61.70 (13.25)	
≥4	22 (7.6)	66.09 (16.44)	
Duration of diabetes, *n* (%)			0.641
≤ 3 months	26 (9.0)	67.42 (14.67)	
>3 months, ≤ 1 year	38 (13.1)	63.21 (14.18)	
>1 year, ≤ 3 years	55 (19.0)	61.78 (14.43)	
>3 years, ≤ 10 years	86 (29.7)	64.05 (15.29)	
>10 years	85 (29.3)	63.73 (15.16)	
Environmental context			
Family Apgar index, Mean (SD)	7.12 (2.39)	63.73 (15.16)	< 0.001^*^
Social support, Mean (SD)	40.22 (8.31)	63.73 (15.16)	< 0.001^*^
Internal resiliency factors			
Self-efficacy, Mean (SD)	25.79 (5.68)	63.73 (15.16)	< 0.001^*^
Depression severity index, Mean (SD)	0.98 (0.87)	63.73 (15.16)	< 0.001^*^
General wellbeing, Mean (SD)	74.44 (12.67)	63.73 (15.16)	< 0.001^*^
Person-environment transactional process			
Confrontation coping, Mean (SD)	19.56 (3.16)	63.73 (15.16)	< 0.001^*^
Surrender coping, Mean (SD)	9.67 (2.82)	63.73 (15.16)	< 0.001^*^
Avoidance coping, Mean (SD)	16.81 (2.47)	63.73 (15.16)	< 0.001^*^

^*^Statistically significant.

### 4.2 Relationship between psychological resilience and socio-demographic variables as well as diabetes-related characteristics

The MLR, as shown in Table 3, demonstrated that level of education, most hobbies, monthly household income of 1,001–2,000 and >3,000, had significant positive relationships with psychological resilience in this study. However, the increase in number of comorbidities or complications had a significantly negative relationship with psychological resilience. These variables jointly explained 13.6% of the variance in resilience, with VIF values (1.263–4.926) indicating no problematic multicollinearity.

**Table 3:** Multiple linear regression of the socio-demographic variables and diabetes related characteristics predicting psychological resilience.

Independent variable	Model 1			
	*B*	*SE B*	β	*P*
Constant	48.21	6.41		< 0.001^*^
Age	0.121	0.08	0.11	0.115
Gender (reference: female)				
Male	0.45	1.88	0.02	0.810
Level of education (reference: primary school or less)				
Junior high school	7.69	2.92	2.63	0.009^*^
Senior high school or secondary technical school	8.89	2.93	3.03	0.003^*^
Junior college	9.00	3.48	2.59	0.010^*^
Undergraduate and above	8.84	3.78	2.34	0.020^*^
Region of residence (reference: urban				
Rural	1.89	2.48	0.06	0.448
Hobbies (reference: few)				
A few	3.02	2.51	0.10	0.230
Moderate	5.84	3.19	0.12	0.068
Most	7.67	3.40	0.17	0.025^*^
Family monthly income per capita (RMB) (reference:<500)				
(500–1,000)	2.15	3.47	0.05	0.536
(1,001–2,000)	8.12	3.84	0.19	0.035^*^
(2,001–3,000)	3.69	3.63	0.11	0.311
>3,000	7.55	3.83	0.24	0.049^*^
Number of co-morbidities or complications (reference: none)				
1	−8.41	2.33	−0.24	< 0.001^*^
2	−3.68	2.68	−0.10	0.171
3	−5.47	2.66	−0.14	0.041^*^
≥4	−2.44	3.70	−0.04	0.510
Duration of diabetes (reference: ≤ 3 months)				
>3 months, ≤ 1 year	−4.33	3.70	−0.10	0.243
>1 year, ≤ 3 years	−5.34	3.49	−0.14	0.127
>3 years, ≤ 10 years	−2.27	3.36	−0.07	0.501
>10 years	−4.31	3.58	−0.13	0.229
*R*^2^ = 0.201, Δ*R*^2^ = 0.136, *F* = 3.062, *P* < .001				

^*^Statistically significant.

### 4.3 Relationship between family Apgar index, social support, self-efficacy, index of depression, general wellbeing, medical coping styles and psychological resilience

Table 4 (Model 2) showed significant positive associations with social support, self-efficacy, and confrontation coping, and negative associations with depression severity index and surrender coping (54.2% variance explained). After further adjustment for socio-demographic and diabetes-related factors, in model 3, social support (β = 0.34), self-efficacy (β = 0.99), depression (β = –29.31), and surrender coping (β = –0.98) remained significant, while confrontation coping became non-significant (*P* = 0.079). The four variables together explained an additional 1.5% of the variance.

**Table 4:** Multiple linear regression of family Apgar index, social support, self-efficacy, index of depression, general wellbeing, medical coping styles predicting psychological resilience.

Variable	Model 2				Model 3			
	*B*	*SE B*	β	*P*	*B*	*SE B*	β	*P*
Constant	39.30	11.28		0.001	38.05	12.57		0.003
Environmental context								
Family Apgar Index	0.31	0.29	0.05	0.284	0.42	0.31	0.07	0.17
Social support	0.33	0.09	0.18	< 0.001^*^	0.34	0.09	0.19	< 0.001^*^
Internal resiliency factors								
Self-efficacy	1.02	0.13	0.38	< 0.001^*^	0.99	0.13	0.37	< 0.001^*^
Depression severity index	−29.61	9.01	−0.20	0.001^*^	−29.31	9.77	−0.19	0.003^*^
General wellbeing	−0.01	0.07	−0.01	0.861	−0.01	0.07	−0.01	0.802
Person-environment transactional process								
Confrontation coping	0.43	0.21	0.09	0.041^*^	0.37	0.21	0.08	0.079
Surrender coping	−0.92	0.29	−0.17	0.002^*^	−0.98	0.30	−0.18	< 0.001^*^
Avoidance coping	−0.03	0.27	−0.01	0.92	−0.07	0.28	−0.01	0.81
	*R*^2^ = 0.554, Δ*R*^2^ = 0.542, *F* = 43.675, *P* < 0.001				*R*^2^ = 0.603, Δ*R*^2^ = 0.557, *F* = 13.123, *P* < 0.001			

Model 2: unadjusted.

Model 3: adjusted for gender, age, level of education, region of residence hobbies, family monthly income per capita, number of co-morbidities or complications, and duration of diabetes.

^*^Statistically significant.

### 4.4 Interview findings

Interviewees were mostly males (*n* = 11, 73.33 %), married (*n* = 14, 93.33 %) and all spoke Chinese (Table 5). Mean age was 54.73 ± 16.66 years and most resided in urban areas (*n* = 13, 86.67 %). Seven (46.67%) reported that they had co-morbidities or complications and the mean duration of diabetes was 10.34 ± 7.92 years. The findings elucidated how 15 respondents coped with their illnesses, adjusted their mental state under stressful situations, and identified the factors that facilitated this process. This was consistent with Kumpfer's resilience framework.

**Table 5:** Demographic and clinical characteristics of the interviewees (*n* = 15).

Participant	Gender	Age (years)	Marital status	Level of education	Region of residence	Family monthly income per capita (RMB)	Number of co-morbidities or complications	Duration of diabetes
P1	Female	66	Married	4	Urban	>3,000	2	16 years
P2	Female	75	Married	2	Urban	>3,000	1	19 years
P3	Male	36	Married	5	Urban	2,001–3,000	0	6 years
P4	Male	81	Married	3	Urban	>3,000	8	22 years
P5	Male	28	Unmarried	5	Urban	>3,000	0	2 years
P6	Male	32	Married	5	Urban	>3,000	0	1 month
P7	Male	57	Married	4	Urban	>3,000	1	4 years
P8	Male	36	Married	4	Urban	>3,000	0	7 years
P9	Male	60	Married	3	Rural	2,001–3,000	0	18 years
P10	Male	63	Married	2	Urban	500–1,000	1	10 years
P11	Male	64	Married	2	Urban	>3,000	0	8 years
P12	Male	36	Married	4	Urban	>3,000	1	4 years
P13	Male	65	Married	3	Urban	>3,000	2	22 years
P14	Female	60	Married	3	Urban	>3,000	0	17 years
P15	Female	62	Married	2	Rural	500–1,000	0	1 month

Level of Education = 1: “Primary school or less,” 2: “Junior high school,” 3: “Senior high school or Secondary technical school,” 4: “Junior college,” 5: “Undergraduate and above.”

### 4.5 Identification of facilitators

Despite various stress, the 15 interviewees still demonstrated a high level of psychological resilience, with scores on the CD-RISC scale exceeding 74 points. Through analysis of the interview data, several factors promoting psychological resilience were identified as shown in Table 6. Thematic analysis identified 10 facilitators—variety of social support, availability of social resources, altruism (participants often expressed that concern for their children's health motivated them to manage their diabetes), diabetes learning journey (participants described that gradually acquiring knowledge and skills about diabetes helped them normalize the condition and reduce distress over time), cognitive reappraisal (including humor-based coping, downward comparison, benefit finding), physiological resilience reserve (participants noted that feeling physically robust, having few complications, or being able to tolerate blood sugar fluctuations helped them maintain a positive outlook) and etc. It further identified the positive association from psychological resilience to self-efficacy.

**Table 6:** Facilitators of psychological resilience in the qualitative research.

Domain of resilience framework	Themes	Sub-theme	Quotations
Environment context	Availability of social resources		“I wasn't really nervous because I knew that hospitals were available and the medical treatments for diabetes are quite good now.” [Participant 8, male] “We had a railway staff hospital that frequently held diabetes health education lectures, and I always attended them. Blood sugar tests were free there.” [Participant 3, male]
	Variety of social support		“The most important factor in helping me recover was my wife's care and reminders (laugh). She helped me learn a lot about this.” [Participant 7, male] “I was very nervous at the time because I didn't understand it at all. The mental burden was heavy, and I lost about 17 pounds in two months. However, after the doctor explained it to me, I felt relieved once I understood.” [Participant 11, male]
Internal resiliency factors	Positive mindset accumulation		“The society now was so good, the medication available was effective, and science was advanced. As long as I followed the doctor's instructions and took my medicine and injections, everything would be fine. I remained very optimistic about it.” [Participant 2, female] “I believed I could handle it. I'd been in the hospital for over ten days, eating that nutrition meal the whole time. I never ate outside. I had confidence in myself.” [Participant 15, female]
	Physiological resilience reserve		“I felt that my condition was pretty good, quite manageable. When I ate more, my blood sugar would go up, and when I ate less, it would come down. It was within the range that I could control, and I didn't feel that it affected my life or mood.” [Participant 6, male] “I never really believed I had diabetes because I didn't have any obvious symptoms. The only thing was that my blood sugar was high; I didn't have the typical ‘three highs and one low' symptoms. So, my mindset stayed pretty positive.” [Participant 7, male]
	Diabetes learning journey		“At first, everything felt very tense because there was a lot I didn't understand. But as I slowly learned more, things felt much more relaxed and less stressful.” [Participant 8, male] “Actually, through my understanding, I realized that for someone like me, not getting this disease would be unusual. I felt it was impossible for me not to have this disease because my habits were really bad.” [Participant 11, male]
	Altruism		“There was not any burden; it was just a matter of taking care of oneself. There was nothing to worry about. The only concern was not letting the kids worry. It was important to take good care of oneself so they would not have to worry.” [Participant 1, female] “I felt it was inevitable for someone like me. My biggest concern was that my daughter wouldn't get this disease. If I wanted to educate our daughter not to do the same thing, I needed to set a good example first.” [Participant 11, male]
Person-environment transactional process	Acceptance-based coping		“Actually, I had many health issues, and I knew they couldn't be cured, so I accepted them peacefully. At my age, these things were inevitable. Getting nervous wouldn't prevent death either.” [Participant 11, male] “My mindset was peaceful. I wasn't careless, but I wasn't overly worried either. I needed to have the right attitude. The condition existed, and the focus was on managing it through diet and exercise.” [Participant 6, male]
	Avoidance-based coping		“I just didn't want to think about getting sick. I only focused on making each day good. I wasn't worried because I was at an age where death was inevitable. If I had to die, I hoped it would be quick and without suffering. I simply did what I enjoyed, like playing and watching things I liked.” [Participant 4, male] “When I felt down, I would play cards. I liked playing cards, and once I started, I wouldn't think about anything else.” [Participant 9, male]
	Cognitive reappraisal	Humor-based coping	“No, I actually felt very fulfilled. I collected a lot of medicine, and I developed a new hobby—collecting medicine. It made me feel very satisfied (laugh).” [Participant 7, male] “My wife didn't allow me to eat sweets, but I would sneak them. Whenever she saw me, she'd say, ‘Confiscated!' and eat them herself. I was left fuming, with nothing left (laugh).” [Participant 12, male]
		Downward comparison	“When I found out, it was only 8.1, which wasn't very high. Some people discover levels in the teens or even twenties, or they've lost a lot of weight. I hadn't, I had just lost a little bit, so I felt it was okay.” [Participant 1, female] “I felt that I still hadn't adapted well enough, mainly because I couldn't control myself with some delicious foods. I needed to work harder on this. I should think about how others quit smoking and get that kind of determination. Thinking about them might make me feel better.” [Participant 10, male]
		Benefit finding	“There was definitely a gain; I came to value health a lot more. Under normal circumstances, a blood sugar level of 6.1 wouldn't affect daily life and work, and wouldn't lead someone to come here and ask for hospitalization like I did” [Participant 3, male] “In the past, I wasn't very careful. If I had done some physical labor, I might not have ended up in this situation. I still needed exercise. Now that I have diabetes, I realized even more how important it is to pay attention to my health.” [Participant 10, male]
	The power of role models		“I didn't think much about it. This was not cancer; I had it for only a few months. There was an elderly lady next door who had diabetes for over 20 years, and she was still fine. She moved to a nursing home, and she seemed to be doing very well.” [Participant 14, female] “Because there was a person near our house who was in particularly good spirits. He had cancer, not diabetes. He even joked with me. I thought he had an especially good attitude. So I wasn't worried.” [Participant 10, male]
Resilience process	Enhance self-efficacy		“Basically, as long as it didn't rain, I could stick to exercising. Every week, I would check my blood sugar. During this period, my blood sugar had some fluctuations. I paid close attention to my diet.” [Participant 7, male] “In terms of exercise, I was diligent. I preferred morning workouts and usually exercised for at least an hour and a half every day. I only skipped if it rained heavily, but that wasn't a problem since I had a treadmill at home.” [Participant 2, female]

### 4.6 Integration

The patients shared their experiences of the psychological resilience process while confronting various stressors and managing diabetes, highlighting the factors that facilitated their recovery to a positive psychological state. In keeping with convergent design in mixed-methods study, the quantitative findings are integrated with the phenomenological interview findings here. Table 7 presents an integrated results matrix where facilitators from participants' experiences were presented against results from the MLR and organized by resilience framework. The integration revealed three patterns:

**Table 7:** Integrated results matrix organized according to resilience framework by (Kumpfer 2002).

Mixed-methods study domains	Promoting factors assessed by the standardized questionnaires		Promoting factors explored through in-depth interviews	Mixed-methods meta-inferences
	Promoting factors	Adjusted^a^ *B* (*SE*), *P*		
Environmental context	Social support	0.34 (0.09), *P* < 0.001^*^	1. Variety of social support 2. Availability of social resources	Converge
Internal resiliency factors	Depression severity index	−29.31 (9.77), *P* < 0.001^*^	1. Positive mindset accumulation 2. Altruism 3. Diabetes learning journey 4. Physiological resilience reserve	Converge Expansion
Person-environment transactional process	Surrender coping	−0.98 (0.30), *P* < 0.001^*^	1. Acceptance-based coping	Diverge
	Avoidance coping	−0.07 (0.28), *P* = 0.81	1. Avoidance-based coping 1. Cognitive reappraisal 2. The power of role models	Diverge Expansion
Resilience process	Self-efficacy	0.99 (0.13), *P* < 0.001^*^	1. Enhance self-efficacy	Diverge

^*^Statistically significant.

Convergence: Social support (quantitative positive) aligned with qualitative themes of the variety of social support and availability of resources. Depression severity (quantitative negative) aligned with qualitative positive mindset accumulation (inverse relationship).

Divergence: Surrender coping (quantitative negative) contrasted with qualitative descriptions of acceptance-based coping as helpful. Avoidance coping (quantitative non-significant) was described by some participants as subjectively helpful.

Expansion: Qualitative data identified several facilitators not measured in the quantitative survey: altruism, diabetes learning journey, physiological resilience reserve, cognitive reappraisal, and the power of role models.

Additionally, while the quantitative regression showed self-efficacy as positively associated with resilience, qualitative participants also described that their resilience helped them persist in diabetes self-management.

## 5 Discussion

This study reveals the complex factors shaping psychological resilience in diabetes care. The convergent design allowed us to identify not only confirmatory patterns but also discrepancies and expansions that would be missed in single-method studies. While social support and self-efficacy strongly promote resilience, depression significantly undermines it. Interestingly, general family-function measures failed to capture the crucial role of spousal support evident in patients' narratives. Additionally, qualitative data added “diabetes learning journey” and “physiological reserve” as internal resilience resources. The research uncovered important nuances in coping strategies: avoidance proved ineffective, while acceptance showed paradoxical effects depending on whether it reflected healthy adaptation or passive resignation. Most notably, the study identified a mutually reinforcing relationship between resilience and self-efficacy, challenging the conventional one-way models.

### 5.1 Facilitators: environmental context

Availability of social resources and social support were external factors that impact the level of psychological resilience. In keeping with previous observations in people with type 1 diabetes, family functioning and interpersonal strategies can promote psychological resilience (Skedgell et al., 2021; Luo et al., 2019b). Social support networks provide important emotional support, information, and material resources for patients (Farrell et al., 2022). These support systems have been shown to promote overall wellbeing, facilitate positive behaviors, and alleviate stress (Gable and Bedrov, 2022). In the qualitative study, some interviewees indicated that their partners would proactively learn about dietary and medication management to better participate in the treatment and recovery of the patients. Therefore, the significance of family resources should not be overlooked, even if not conclusively confirmed by quantitative research. Facilitating communication and intimate relationships with others in one's social network, known as “build your connections,” typically leads to the expansion of positive and emotionally meaningful sources of social support (Southwick et al., 2005). These skills should be incorporated into intervention promoting psychological resilience in patients to cope with the burdens or challenges related to diabetes. Assessing the extent of a patient's social support network, as well as the level of emotional support they receive, is crucial when providing therapy.

### 5.2 Facilitators: internal resiliency factors

The depression severity index and positive mindset accumulation were factors associated with psychological resilience in people with diabetes. Specifically, the depression severity index was negatively associated with resilience (Wojujutari et al., 2019), corresponding to positive mindset identified in qualitative studies. In a study of American people with diabetes, a strong negative correlation was found between depression and resilience (Olson et al., 2023). Conversely, a regression analysis indicated that optimism could predict an improvement in the ability to broad-minded coping (Fredrickson and Joiner, 2002), and improved coping subsequently enhances resilience (Foster et al., 2019). When depression and positive emotions coexist, Folkman proposed a hypothesis that, the positive emotions may provide psychological rest and replenish depleted resources consumed by stress (Folkman and Moskowitz, 2000). Thus, cognitive-behavioral therapy can be employed to teach individuals how to adjust negative thought patterns and foster a more positive perspective.

Altruism was another important facilitator which help people develop psychological resilience undergoing diabetes. Influenced by traditional Chinese culture, the majority of people with diabetes in this study indicated that they did not have religious beliefs. Considering the wellbeing of children is a source of their emotional strength, which is another form of altruism—finding meaning through contributing to the welfare of children (Caviola et al., 2021). A qualitative study involving nurses showed that conveying altruism helps cultivate psychological resilience in the workplace (Wei et al., 2019). However, this facilitating factor has not been directly verified in this study. Future research could develop an altruism scale suitable for Chinese culture to further validate it.

The interview results supplemented the facilitators not covered in quantitative research: diabetes learning journey. It includes experiences with managing high blood sugar and knowledge about the daily management. This helps individuals recognize that diabetes is manageable, thereby maintaining psychological balance. Many challenges encountered in the daily diabetes management require careful calculations and decision-making based on anticipated consequences, all of which involve complex thought processes (Hilliard et al., 2012). Gradual accumulation of knowledge and skills aid patients in managing diabetes daily through effective problem-solving in adversity, gradually boosting psychological resilience to cope with challenges (Berg et al., 2011; Hughes et al., 2012). Personalized education plans (with smart assessment of patients' age, educational level, and disease) and technology-assisted approaches (video consultations, online appointments) will provide effective pathway.

Physiological resilience reserve was also a promoting factor which was somewhat associated with the number of co-morbidities or complications. Physiological resilience reserve refers to the body's ability to cope with challenges and stress through its own regulatory and adaptive mechanisms. When this reserve is sufficient, patients typically have greater stress coping ability and emotional stability when facing diabetes. A study on Physical Resilience and Aging shows that physiological resilience in older adults may enhance their psychological coping abilities in response to clinical stressors (Walston et al., 2023). The number of comorbidities and complications acts as a stressor that impedes the development of resilience, posing a threat to individuals' coping abilities (Ghanei Gheshlagh et al., 2016). Physiological resilience is a newly proposed concept, and its definition and measurement need further precision and refinement (Colon-Emeric et al., 2023).

### 5.3 Facilitators: person-environment transactional process

Respondents' interviews suggest that acceptance-based and avoidance-based coping could promote psychological resilience. However, in quantitative studies, yielding coping (also known as acceptance) has a negative impact on psychological resilience, and avoidance coping shows no significant effect.

Passively accepting reality implies surrendering to the adversity. Many highly resilient individuals believe that acceptance is the key trait that enables them to withstand highly stressful environments (Southwick et al., 2005). A review also indicated that acceptance was associated with greater psychological adjustment following trauma (Thompson et al., 2011). While acceptance is not an active coping strategy to address sources of stress, it is the most common response, which predicts less distress (Carver et al., 1993). In some cases, excessive acceptance may cause patients to lose motivation to improve their diabetes condition, leading them to abandon treatment or rehabilitation plans, thus negatively affecting their psychological resilience. Investigating the factors influencing surrender coping (acceptance-based coping), specifically the patients' circumstances, is the next step.

Although in the short term, avoidance-based coping strategies may seem effective in avoiding direct confrontation with negative emotions (Schreiber and Richards, 2024), this strategy is like throwing a boomerang: although it temporarily moves away after being thrown, it eventually returns to the thrower. Similarly, long-term avoidance of difficulties makes problems more challenging (Straton et al., 2024; Sege et al., 2018). The long-term negative impacts of this coping strategy highlight its limitations in psychological adaptation and problem-solving ability. It should be noted that the MCMQ showed modest internal consistency (Cronbach's α = 0.498) in this sample, so the quantitative estimates for coping styles should be interpreted with some caution. Quantitatively, the four variables (social support, self-efficacy, depression, and surrender coping) together explained an additional 1.5% of the variance in resilience, suggesting they are strongly associated with resilience, with limited additional contribution from other covariates. The finding that confrontation coping was no longer significant after adjustment hints that demographic factors such as education or income may partially account for its apparent association.

Cognitive reappraisal is a cognitive strategy for building psychological resilience, including humor coping, downward comparison, and benefit finding. Humor is considered an important coping mechanism that can reduce the threat of stressful situations through cognitive reappraisal (Culver et al., 2002), as well as alleviate negative emotions by attracting social support (Silver et al., 1990). Downward comparison and benefit finding (Affleck and Tennen, 1996; Kedia et al., 2013), both are cognitive coping strategies used to reframe a situation, enabling individuals to positively reassess difficult circumstances. The ability to cognitively reevaluate, reframe, or find positive meaning in adverse events is a characteristic of many resilient individuals (Xin et al., 2023). In Janoff-Bulman's model, cognitive reappraisal in the face of adversity may involve a greater appreciation of existing strengths (e.g., self-discipline), the development of admirable qualities (e.g., wisdom), and an awareness of the preciousness of life or health (Janoff-Bulman, 1989). In cognitive behavioral therapy, positive reevaluation is often taught and encouraged. Healthcare professionals should convey a value that believes one can live a “normal” life with diabetes, look for benefits, and view diabetes management as an integral part of life.

The power of role model plays a crucial role in the education and development of individuals. From resilient role models, people can acquire better skills for coping with life's challenges (Mealer et al., 2012). Mealer et al. demonstrated through qualitative research that resilient role models help nurses manage stress in their work environment (Mealer et al., 2012). Quantitative studies have shown that having non-parental role models can promote psychological resilience in school children (Isumi et al., 2023). These findings indicate that previous research has established a link between successful role models and psychological resilience. This study further elucidates the underlying reasons for this association by exploring the experiences of people with diabetes. Healthcare professionals can invite resilient patients to share their experiences, providing peer support to correct erroneous cognitions and negative coping strategies, thereby building confidence.

### 5.4 Resilience process: enhance self-efficacy

Quantitative research shows that self-efficacy is a promoting factor of psychological resilience, but qualitative research suggests that enhanced resilience can increase self-efficacy. The inconsistency in findings may suggest that psychological resilience and self-efficacy are mutually reinforcing factors. A cross-sectional study of patients with type 2 diabetes, from a Taiwan clinic, showed that improving psychological resilience enhances self-efficacy, but has no effect in highly distressed patients (Wang et al., 2023). A structural equation model indicated that self-efficacy directly or indirectly improves the stress faced by undergraduate students when dealing with environmental difficulties (Bodys-Cupak et al., 2016). Its success can be driven by health education promoting the maintenance of healthy habits (Ferreira et al., 2022). When promoting health education, it is necessary to consider the interaction between self-efficacy and psychological resilience.

## 6 Limitations

This study employed a theory-driven approach and a convergent mixed-methods design to conduct an in-depth investigation of the factors influencing psychological resilience in patients with diabetes. However, there are also some limitations to consider. Participants included in qualitative research were predominantly males, married and living in urban areas, limiting representativeness. Future research should ensure a more diverse and representative sample, particularly in rural areas and among women. Given the cross-sectional nature of the quantitative analysis, the results should be interpreted cautiously. Although self-reporting is widely used in the literature, this data collection method is characterized by a high level of subjectivity, inevitably leading to some biases in the data. Additionally, the qualitative sample was limited to highly resilient participants (CD-RISC >74), which may not represent the full spectrum of adaptation.

## 7 Conclusion

Optimizing facilitators may promote psychological resilience among people with diabetes. Joint efforts should be made by relevant stakeholders to expand social support networks and ensure equitable access to resources, while empowering patients through positive mindset cultivation and cognitive-reappraisal techniques such as humor and benefit-finding. Motivational strategies grounded in altruism—for example, setting family health goals—should be routinely incorporated. Special attention must be paid to distinguishing adaptive acceptance from passive surrender in coping processes. In clinical practice, clinicians and health psychologists should embed dynamic monitoring tools that capture the dual effects of acceptance coping and the bidirectional associations between resilience and self-efficacy; systematically integrating these approaches into chronic-disease health education can simultaneously improve mental health and disease-management behaviors. Future research ought to develop and test population-specific interventions that effectively bolster these facilitators, with special attention to high-need groups such as diabetic youth and socioeconomically marginalized individuals.

## Data Availability

The raw data supporting the conclusions of this article will be made available by the authors, without undue reservation.

## References

[B1] Affleck G. Tennen H. (1996). Construing benefits from adversity: adaptational significance and dispositional underpinnings. J. Pers. 64, 899–922. doi: 10.1111/j.1467-6494.1996.tb00948.x 8956517

[B2] Anderson K. Priebe S. (2021). Concepts of resilience in adolescent mental health research. J. Adolesc. Health 69, 689–695. doi: 10.1016/j.jadohealth.2021.03.035 34045094

[B3] Avogaro A. Fadini G. P. (2019). Microvascular complications in diabetes: a growing concern for cardiologists. Int. J. Cardiol. 291, 29–35. doi: 10.1016/j.ijcard.2019.02.030 30833106

[B4] Berg C. A. King P. S. Butler J. M. Pham P. Palmer D. Wiebe D. J. . (2011). Parental involvement and adolescents' diabetes management: the mediating role of self-efficacy and externalizing and internalizing behaviors. J. Pediatr. Psychol. 36, 329–339. doi: 10.1093/jpepsy/jsq088 20926405 PMC3062285

[B5] Bodys-Cupak I. Majda A. Zalewska-Puchała J. Kamińska A. (2016). The impact of a sense of self-efficacy on the level of stress and the ways of coping with difficult situations in Polish nursing students. Nurse Educ. Today 45, 102–107. doi: 10.1016/j.nedt.2016.07.004 27429414

[B6] Bohler F. Bohler L. Taranikanti V. (2024). Targeting pericyte retention in diabetic retinopathy: a review. Ann. Med. 56:2398200. doi: 10.1080/07853890.2024.2398200 39268600 PMC11404372

[B7] Carver C. S. Pozo C. Harris S. D. Noriega V. Scheier M. F. Robinson D. S. . (1993). How coping mediates the effect of optimism on distress: a study of women with early stage breast cancer. J. Pers. Soc. Psychol. 65, 375–390. doi: 10.1037/0022-3514.65.2.375 8366426

[B8] Caviola L. Schubert S. Greene J. D. (2021). The psychology of (in)effective altruism. Trends Cogn. Sci. 25, 596–607. doi: 10.1016/j.tics.2021.03.015 33962844

[B9] Chew B. H. Vos R. C. Metzendorf M. I. Scholten R. J. Rutten G. E. (2017). Psychological interventions for diabetes-related distress in adults with type 2 diabetes mellitus. Cochrane Database Syst. Rev. 9:Cd011469. doi: 10.1002/14651858.CD011469.pub2 28954185 PMC6483710

[B10] Colaizzi P. F. (1978). “Psychological research as the phenomenologist views it,” in Existential-Phenomenological Alternatives for Psychology, eds. R. S. Valle and M. King (New York: Oxford University Press), 48–71.

[B11] Colon-Emeric C. Schmader K. Cohen H. J. Morey M. Whitson H. (2023). Ageing and physical resilience after health stressors. Stress Health 39, 48–54. doi: 10.1002/smi.3241 36879359 PMC10480330

[B12] Connor K. M. Davidson J. R. (2003). Development of a new resilience scale: the Connor-Davidson resilience scale (CD-RISC). Depression Anxiety 18, 76–82. doi: 10.1002/da.10113 12964174

[B13] Creswell J. W. Creswell J. D. (2018). Research Design: Qualitative, Quantitative, and Mixed Methods Approaches, 5th Edn. Thousand Oaks, CA: SAGE Publications.

[B14] Creswell J. W. Poth C. N. (2018). Qualitative Inquiry and Research Design: Choosing Among Five Approaches, 4th Edn. Thousand Oaks, CA: SAGE Publications.

[B15] Culver J. L. Arena P. L. Antoni M. H. Carver C. S. (2002). Coping and distress among women under treatment for early stage breast cancer: comparing African Americans, Hispanics and non-Hispanic Whites. Psychooncology 11, 495–504. doi: 10.1002/pon.615 12476431

[B16] Dai Q. Kyuragi Y. Zakia H. Oishi N. Yao L. Zhang Z. . (2024). Psychological resilience is positively correlated with Habenula volume. J. Affect. Disord. 365, 178–184. doi: 10.1016/j.jad.2024.08.012 39151760

[B17] Farrell A. K. Stanton S. C. E. Marshall E. M. (2022). Social network structure and combating social disconnection: implications for physical health. Curr. Opin. Psychol. 45:101313. doi: 10.1016/j.copsyc.2022.101313 35338892

[B18] Feifel H. Strack S. Nagy V. T. (1987). Coping strategies and associated features of medically ill patients. Psychosom. Med. 49, 616–625. doi: 10.1097/00006842-198711000-00007 3423168

[B19] Ferreira M. A. Belchior A. B. Alencar C. S. Almeida P. C. Nascimento F. G. Oliveira S. K. P. . (2022). Resilience of people with diabetes mellitus during the COVID-19 pandemic. Rev. Gaucha Enferm. 43:e20210202. doi: 10.1590/1983-1447.2022.20210202.en 36383817

[B20] Folkman S. Moskowitz J. T. (2000). Positive affect and the other side of coping. Am. Psychol. 55, 647–654. doi: 10.1037/0003-066X.55.6.647 10892207

[B21] Foster K. Roche M. Delgado C. Cuzzillo C. Giandinoto J. A. Furness T. . (2019). Resilience and mental health nursing: an integrative review of international literature. Int. J. Ment. Health Nurs. 28, 71–85. doi: 10.1111/inm.12548 30294937

[B22] Fredrickson B. L. Joiner T. (2002). Positive emotions trigger upward spirals toward emotional well-being. Psychol. Sci. 13, 172–175. doi: 10.1111/1467-9280.00431 11934003

[B23] Gable S. L. Bedrov A. (2022). Social isolation and social support in good times and bad times. Curr. Opin. Psychol. 44, 89–93. doi: 10.1016/j.copsyc.2021.08.027 34600413

[B24] Genitsaridi I. Salpea P. Salim A. Sajjadi S. F. Tomic D. James S. . (2026). 11th edition of the IDF diabetes atlas: global, regional, and national diabetes prevalence estimates for 2024 and projections for 2050. Lancet Diabetes Endocrinol. 14, 149–156. doi: 10.1016/S2213-8587(25)00299-2 41412135

[B25] Ghanei Gheshlagh R. Sayehmiri K. Ebadi A. Dalvandi A. Dalvand S. Nourozi Tabrizi K. . (2016). Resilience of patients with chronic physical diseases: a systematic review and meta-analysis. Iran. Red Crescent Med. J. 18:e38562. doi: 10.5812/ircmj.38562 27703800 PMC5027800

[B26] Guetterman T. C. Fetters M. D. Creswell J. W. (2015). Integrating quantitative and qualitative results in health science mixed methods research through joint displays. Ann. Fam. Med. 13, 554–561. doi: 10.1370/afm.1865 26553895 PMC4639381

[B27] Guo H. Zhou R. Li M. Zhang S. Yi H. Wang L. . (2022). The use of Kumpfer's resilience framework in understanding the breastfeeding experience of employed mothers after returning to work: a qualitative study in China. Int. Breastfeed J. 17:13. doi: 10.1186/s13006-022-00459-8 35193604 PMC8864792

[B28] Hessler D. M. Fisher L. Polonsky W. H. Masharani U. Strycker L. A. Peters A. L. . (2017). Diabetes distress is linked with worsening diabetes management over time in adults with Type 1 diabetes. Diabet. Med. 34, 1228–1234. doi: 10.1111/dme.13381 28498610 PMC5561505

[B29] Hilliard M. E. Harris M. A. Weissberg-Benchell J. (2012). Diabetes resilience: a model of risk and protection in type 1 diabetes. Curr. Diab. Rep. 12, 739–748. doi: 10.1007/s11892-012-0314-3 22956459

[B30] Hoogendoorn C. J. Krause-Steinrauf H. Uschner D. Wen H. Presley C. A. Legowski E. A. . (2024). Emotional distress predicts reduced type 2 diabetes treatment adherence in the glycemia reduction approaches in diabetes: a comparative effectiveness study (GRADE). Diabetes Care 47, 629–637. doi: 10.2337/dc23-1401 38227900 PMC10973907

[B31] Hughes A. E. Berg C. A. Wiebe D. J. (2012). Emotional processing and self-control in adolescents with type 1 diabetes. J. Pediatr. Psychol. 37, 925–934. doi: 10.1093/jpepsy/jss062 22523404 PMC3437683

[B32] Isumi A. Doi S. Ochi M. Kato T. Fujiwara T. (2023). School- and community-level protective factors for resilience among chronically maltreated children in Japan. Soc. Psychiatry Psychiatr. Epidemiol. 58, 477–488. doi: 10.1007/s00127-022-02322-x 35842522

[B33] Janoff-Bulman R. (1989). Assumptive worlds and the stress of traumatic events: applications of the schema construct. Social Cogn. 7, 113–136. doi: 10.1521/soco.1989.7.2.113

[B34] Jia J. Jenkins A. J. Quintiliani L. M. Truong V. Lasser K. E. (2022). Resilience and diabetes self-management among African-American men receiving primary care at an urban safety-net hospital: a cross-sectional survey. Ethn. Health 27, 1178–1187. doi: 10.1080/13557858.2020.1849566 33249921

[B35] Joyce S. Shand F. Tighe J. Laurent S. J. Bryant R. A. Harvey S. B. . (2018). Road to resilience: a systematic review and meta-analysis of resilience training programmes and interventions. BMJ Open 8:e017858. doi: 10.1136/bmjopen-2017-017858 29903782 PMC6009510

[B36] Kedia G. Lindner M. Mussweiler T. Ihssen N. Linden D. E. (2013). Brain networks of social comparison. Neuroreport 24, 259–264. doi: 10.1097/WNR.0b013e32835f2069 23407275

[B37] Kumpfer K. L. (2002). “Factors and processes contributing to resilience,” in Resilience and Development. Longitudinal Research in the Social and Behavioral Sciences: An Interdisciplinary Series, eds. M. D. Glantz, J. L. Johnson (Boston, MA: Springer). doi: 10.1007/0-306-47167-1_9

[B38] Kunzler A. M. Helmreich I. König J. Chmitorz A. Wessa M. Binder H. . (2020). Psychological interventions to foster resilience in healthcare students. Cochrane Database Syst. Rev. 7:Cd013684. doi: 10.1002/14651858.CD013684 32691879 PMC7388680

[B39] Kusnanto K. Arifin H. Widyawati I. Y. (2020). A qualitative study exploring diabetes resilience among adults with regulated type 2 diabetes mellitus. Diabetes Metab. Syndr. 14, 1681–1687. doi: 10.1016/j.dsx.2020.08.035 32905940

[B40] Linz S. Helmreich I. Kunzler A. Chmitorz A. Lieb K. Kubiak T. . (2020). Interventions to promote resilience in adults - a narrative review. Psychother. Psychosom. Med. Psychol. 70, 11–21. doi: 10.1055/a-0830-4745 31163455

[B41] Lukács A. Mayer K. Sasvári P. Barkai L. (2018). Health-related quality of life of adolescents with type 1 diabetes in the context of resilience. Pediatr. Diabetes 19, 1481–1486. doi: 10.1111/pedi.12769 30203556

[B42] Luo D. Lin Z. Shang X. C. Li S. (2019a). “I can fight it!”: a qualitative study of resilience in people with inflammatory bowel disease. Int. J. Nurs. Sci. 6, 127–133. doi: 10.1016/j.ijnss.2018.12.008 31406881 PMC6608668

[B43] Luo D. Xu J. J. Cai X. Zhu M. Wang H. Yan D. . (2019b). The effects of family functioning and resilience on self-management and glycaemic control among youth with type 1 diabetes. J. Clin. Nurs. 28, 4478–4487. doi: 10.1111/jocn.15033 31410916

[B44] Mealer M. Jones J. Moss M. (2012). A qualitative study of resilience and posttraumatic stress disorder in United States ICU nurses. Intensive Care Med. 38, 1445–1451. doi: 10.1007/s00134-012-2600-6 22618093

[B45] Mei Y. Yang X. Gui J. Li Y. Zhang X. Wang Y. . (2023). The relationship between psychological resilience and quality of life among the Chinese diabetes patients: the mediating role of stigma and the moderating role of empowerment. BMC Public Health 23:2043. doi: 10.1186/s12889-023-16927-7 37858079 PMC10585926

[B46] Moeller A. J. Creswell J. W. Saville N. (2016). Second *Language Assessment and Mixed Methods Research*. Cambridge: Cambridge University Press.

[B47] Olson K. L. Howard M. McCaffery J. M. Dutton G. R. Espeland M. A. Simpson F. R. . (2023). Psychological resilience in older adults with type 2 diabetes from the Look AHEAD Trial. J. Am. Geriatr. Soc. 71, 206–213. doi: 10.1111/jgs.17986 36196673 PMC9944500

[B48] Ong K. L. Stafford L. K. McLaughlin S. A. Boyko E. J. Vollset S. E. Smith A. E. . (2023). Global, regional, and national burden of diabetes from 1990 to 2021, with projections of prevalence to 2050: a systematic analysis for the Global Burden of Disease Study 2021. Lancet 402, 203–234. doi: 10.1016/S0140-6736(23)01301-6 37356446 PMC10364581

[B49] Östlund U. Kidd L. Wengström Y. Rowa-Dewar N. (2011). Combining qualitative and quantitative research within mixed method research designs: a methodological review. Int. J. Nurs. Stud. 48, 369–383. doi: 10.1016/j.ijnurstu.2010.10.005 21084086 PMC7094322

[B50] Palinkas L. A. Mendon S. J. Hamilton A. B. (2019). Innovations in mixed methods evaluations. Annu. Rev. Public Health 40, 423–442. doi: 10.1146/annurev-publhealth-040218-044215 30633710 PMC6501787

[B51] Park C. S. Choi Y. J. Rhee T. M. Lee H. J. Lee H. S. Park J. B. . (2022). U-shaped associations between body weight changes and major cardiovascular events in type 2 diabetes mellitus: a longitudinal follow-up study of a nationwide cohort of over 1.5 million. Diabetes Care 45, 1239–1246. doi: 10.2337/dc21-2299 35263435

[B52] Perrin N. E. Davies M. J. Robertson N. Snoek F. J. Khunti K. (2017). The prevalence of diabetes-specific emotional distress in people with Type 2 diabetes: a systematic review and meta-analysis. Diabet. Med. 34, 1508–1520. doi: 10.1111/dme.13448 28799294

[B53] Pesantes M. A. Lazo-Porras M. Abu Dabrh A. M. Ávila-Ramírez J. R. Caycho M. Villamonte G. Y. . (2015). Resilience in vulnerable populations with type 2 diabetes mellitus and hypertension: a systematic review and meta-analysis. Can. J. Cardiol. 31, 1180–1188. doi: 10.1016/j.cjca.2015.06.003 26239007 PMC4556590

[B54] Pouwer F. Snoek F. J. van der Ploeg H. M. Adèr H. J. Heine R. J. (2000). The well-being questionnaire: evidence for a three-factor structure with 12 items (W-BQ12). Psychol. Med. 30, 455–462. doi: 10.1017/S0033291700001719 10824665

[B55] Powers B. A. Knapp T. R. (2006). A Dictionary of Nursing Theory and Research, 3rd Edn. New York, NY: Sage Publications.

[B56] Rivera-Picón C. Benavente-Cuesta M. H. Quevedo-Aguado M. P. Juárez-Vela R. Martinez-Tofe J. Sánchez-González J. L. . (2023). Influence of state of health and personality factors of resilience and coping in healthy subjects and those with diabetes. Front. Public Health 11:1074613. doi: 10.3389/fpubh.2023.1074613 36935663 PMC10017435

[B57] Sachar A. Willis T. Basudev N. (2020). Mental health in diabetes: can't afford to address the service gaps or can't afford not to? Br. J. Gen. Pract. 70, 6–7. doi: 10.3399/bjgp20X707261 31879288 PMC6919506

[B58] Schreiber J. Richards M. C. (2024). The cost of avoidance. J. Am. Acad. Child. Adolesc. Psychiatry 63:561. doi: 10.1016/j.jaac.2024.02.004 38387792

[B59] Schwarzer R. Jerusalem M. (2010). The general self-efficacy scale (GSE). Anxiety Stress Coping 12, 329–345. doi: 10.1080/10615809908248330

[B60] Sege C. T. Bradley M. M. Lang P. J. (2018). Avoidance and escape: defensive reactivity and trait anxiety. Behav. Res. Ther. 104, 62–68. doi: 10.1016/j.brat.2018.03.002 29549752 PMC5903567

[B61] Silver R. C. Wortman C. B. Crofton C. (1990). “The role of coping in support provision: the self-presentational dilemma of victims of life crises,” in Social Support: An Interactional View, Wiley Series on Personality Processes, eds. B. R. Sarason, I. G. Sarason, and G. R. Pierce (Oxford: John Wiley and Sons), 397–426.

[B62] Skedgell K. K. Cao V. T. Gallagher K. A. Anderson B. J. Hilliard M. E. (2021). Defining features of diabetes resilience in emerging adults with type 1 diabetes. Pediatr. Diabetes 22, 345–353. doi: 10.1111/pedi.13136 33034097 PMC12168199

[B63] Southwick S. M. Vythilingam M. Charney D. S. (2005). The psychobiology of depression and resilience to stress: implications for prevention and treatment. Annu. Rev. Clin. Psychol. 1, 255–291. doi: 10.1146/annurev.clinpsy.1.102803.143948 17716089

[B64] Straton E. Anifowoshe K. Moore H. Streisand R. Jaser S. S. (2024). Associations of coping strategies with glycemic and psychosocial outcomes among adolescents with type 1 diabetes experiencing diabetes distress. Ann. Behav. Med. 58, 628–633. doi: 10.1093/abm/kaae028 39014980 PMC11305127

[B65] Thompson R. W. Arnkoff D. B. Glass C. R. (2011). Conceptualizing mindfulness and acceptance as components of psychological resilience to trauma. Trauma Violence Abuse 12, 220–235. doi: 10.1177/1524838011416375 21908440

[B66] UK D (2019). Too Often Missing: Making Emotional and Psychological Support Routine in Diabetes Care. London: UK D.

[B67] Walston J. Varadhan R. Xue Q. L. Buta B. Sieber F. Oni J. . (2023). A study of physical resilience and aging (SPRING): conceptual framework, rationale, and study design. J. Am. Geriatr. Soc. 71, 2393–2405. doi: 10.1111/jgs.18483 37386913 PMC10608799

[B68] Wang R. H. Chen S. Y. Lee C. M. Lu C. H. Hsu H. C. (2023). Resilience, self-efficacy and diabetes distress on self-management behaviours in patients newly diagnosed with type 2 diabetes: a moderated mediation analysis. J. Adv. Nurs. 79, 215–222. doi: 10.1111/jan.15483 36317455

[B69] Wei H. Roberts P. Strickler J. Corbett R. W. (2019). Nurse leaders' strategies to foster nurse resilience. J. Nurs. Manag. 27, 681–687. doi: 10.1111/jonm.12736 30449038

[B70] Wojujutari A. K. Alabi O. T. Emmanuel I. E. (2019). Psychological resilience moderates influence of depression on sleep dysfunction of people living with diabetes. J. Diabetes Metab. Disord. 18, 429–436. doi: 10.1007/s40200-019-00436-9 31890668 PMC6915166

[B71] Wu Y. Zhang Y. Y. Zhang Y. T. Zhang H. J. Long T. X. Zhang Q. . (2023). Effectiveness of resilience-promoting interventions in adolescents with diabetes mellitus: a systematic review and meta-analysis. World J. Pediatr. 19, 323–339. doi: 10.1007/s12519-022-00666-7 36534296 PMC9761642

[B72] Xiao S. (1994). The theoretical basis and research application of the social support rating scale. J. Clin. Psychiatry 4, 98–100.

[B73] Xin T. Fang L. Zhang Y. Yang X. Liu W. Chen N. . (2023). Effects of cognitive reappraisal on directed forgetting of negative emotional memory: an ERP study. Psychol. Res. 87, 2146–2157. doi: 10.1007/s00426-023-01811-1 36905453

[B74] Yi-Frazier J. P. Hilliard M. E. O'Donnell M. B. Zhou C. Ellisor B. M. Garcia Perez S. . (2024). Promoting resilience in stress management for adolescents with type 1 diabetes: a randomized clinical trial. JAMA Netw. Open 7:e2428287. doi: 10.1001/jamanetworkopen.2024.28287 39158914 PMC11333977

[B75] Yu Y. Li D. Xia Y. (2024). Applying Kumpfer's resilience framework to understand the social adaptation process of the trailing parents in China. BMC Geriatr. 24:587. doi: 10.1186/s12877-024-05170-3 38982345 PMC11232334

[B76] Zhang Z. (2005). Handbook of Behavioral Medicine Scales. Beijing: Chinese Medical Multimedia Press.

[B77] Zung W. W. (1965). A self-rating depression scale. Arch. General Psychiatry 12, 63–70. doi: 10.1001/archpsyc.1965.01720310065008 14221692

